# Flagellate-like dermatitis in a child with autoinflammatory disorder managed with colchicine

**DOI:** 10.1016/j.jdcr.2022.07.012

**Published:** 2022-07-16

**Authors:** Aya Al Rawahi, Buthaina Al Musalhi, Reem Abdwani

**Affiliations:** a Dermatology Residency Training Program, Oman Medical Specialty Board, Muscat, Sultanate of Oman; b Pediatric Dermatology Unit, Sultan Qaboos University Hospital, Muscat, Sultanate of Oman; c Department of Child Health, College of Medicine and Health Sciences, Sultan Qaboos University, Muscat, Sultanate of Oman

**Keywords:** case report, colchicine, flagellate dermatitis

## Introduction

Flagellate dermatitis is a rare patterned-skin eruption characterized by linear erythematous streaks with hyperpigmentation.^1^ Bleomycin, a sulfur-containing antineoplastic antibiotic, is known to have this side effect.^1^ It has also been reported after the ingestion of raw or undercooked shiitake mushrooms. Recently, a few reports have associated it with autoimmune diseases, such as dermatomyositis, adult-onset Still disease, and systemic-onset juvenile idiopathic arthritis.^1^ Mostly, the skin eruption resolves spontaneously, but in some cases, treatment with antihistamines and corticosteroids (topical or oral) may be required. Here, we present the case of a child with an autoinflammatory disorder who presented with “flagellate-like lesions” after the commencement of anakinra and was managed with colchicine.

## Case report

At the age of 3 months, the patient presented with recurrent episodes of high-grade fever, diffuse urticarial skin eruption, and polyarthritis associated with anemia, leukocytosis, and high-inflammatory markers. She underwent extensive workup for infectious causes, hemophagocytic lymphohistiocytosis, and malignancy, all of which were excluded. In view of the presentation in early infancy of urticarial eruption, the persistence of fever, and extensive polyarthritis (documented by whole-body magnetic resonance imaging), the possibility of an autoinflammatory disorder, such as neonatal-onset multisystem inflammatory disease (NOMID), was considered. An autoinflammatory gene panel showed a heterozygous mutation of Met694 Val in the *MEFV* gene; however, the early presentation did not correlate with the genotype. Further, sequencing at a higher coverage rate was done and the results showed 5% mosaicism in *NLRP3* (p. Asp305Glu), supporting the clinical diagnosis of NOMID.

**Fig 1:**
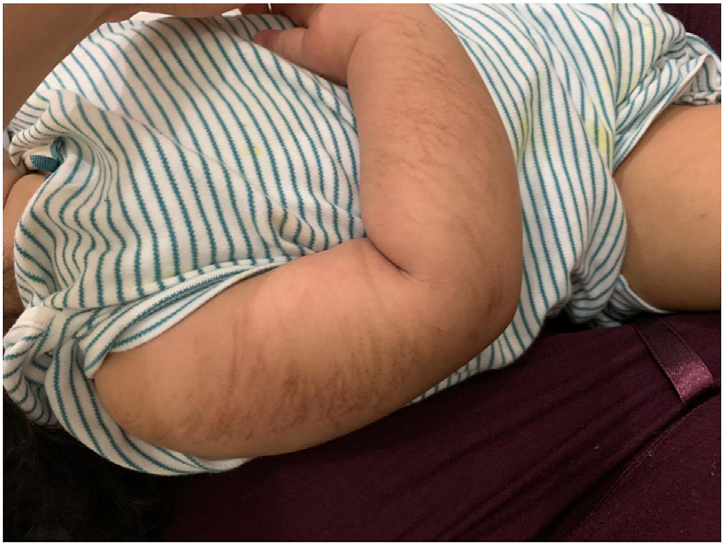
Multiple linear hyperpigmented plaques on the right arm. Similar lesions were present at the same time on the left arm.

**Fig 2:**
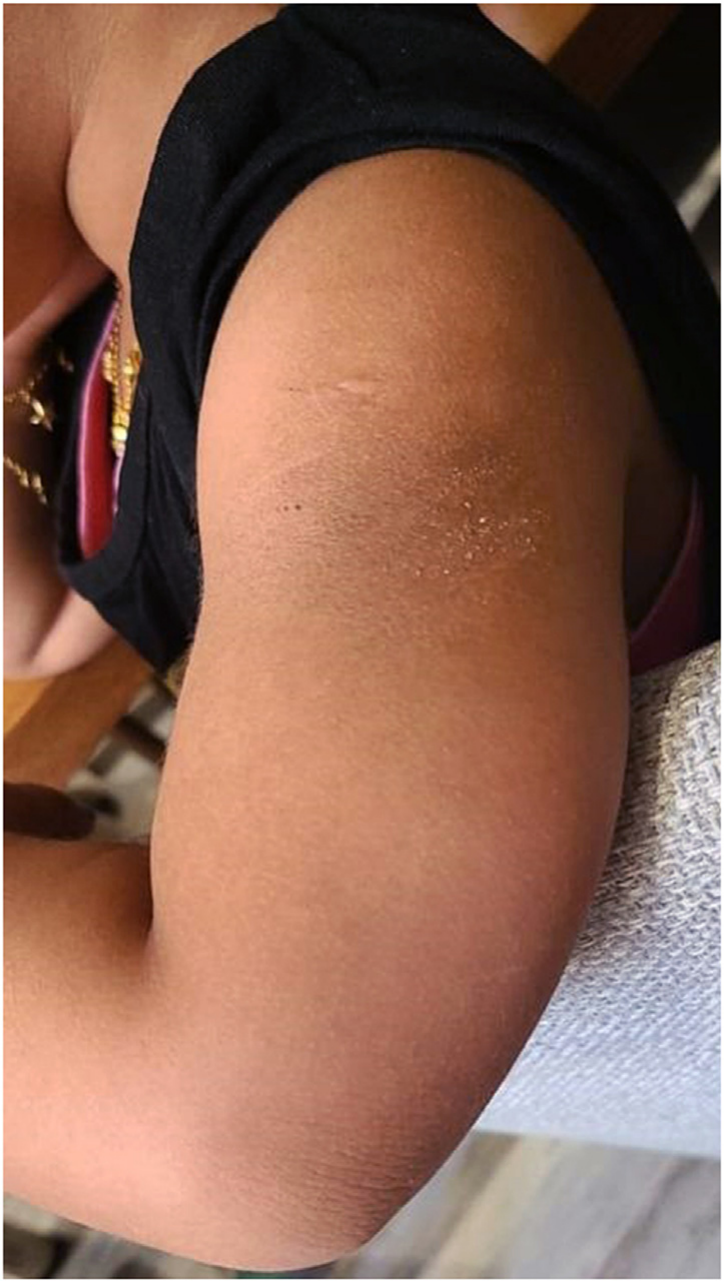
Hyperpigmented patch with dry scales after 3 months from the initiation of colchicine of the left arm.

The patient presented to a dermatology clinic with intermittent, pruritic, erythematous, linear patches, described as “scratch marks”-like lesions, over the back, upper portion of the arms, and thighs (Fig 1). Three months after starting anakinra to treat her primary disease, the eruption appeared. Fever and arthritis had improved after starting anakinra dose at 2 mg/kg, which was later increased to 4 mg/kg, as she outgrew the initial dose. However, the increase in dose aggravated the linear eruption; initially, mid-potency topical corticosteroids were started, but the symptoms did not improve. Colchicine 0.25 mg/d once daily was started to control systemic flares and prevent amyloidosis, then increased to 0.5 mg/d. In addition, the skin lesions improved within a week and the duration of the cutaneous flares was significantly reduced. The patient was followed-up for 15 months; the rash improved dramatically after starting colchicine, with no reported flares (Fig 2).

## Discussion

Flagellate dermatitis presents as erythematous papules, macules, or bullae, in an intermingled linear pattern distributed over the trunk, extremities, and face, and is rarely observed in the pediatric age group. It is highly characteristic of bleomycin treatment but has also been reported with other chemotherapeutic agents such as peplomycin, docetaxel, bendamustine, and doxorubicin.^2^ Shiitake dermatitis is the second most common cause of flagellate dermatitis, occurring after ingestion of uncooked shiitake mushrooms. A few cases of parvovirus 19 infection, chikungunya fever, and mycoplasma pneumonia have been reported.^3^ A similar linear cutaneous eruption has been associated with dermatomyositis and adult-onset Still disease.^1^ Flagellated dermatitis has been recently documented as an atypical cutaneous eruption in SoJIA, described as a persistent, dusky red to brownish plaques, in a linear configuration, associated with fever and musculoskeletal pain.^4^ Systemic-onset juvenile idiopathic arthritis was the main differential diagnosis in our case given the similar clinical picture; however, genetic analysis confirmed the diagnosis of NOMID and Familial Mediterranean Fever (FMF). The patient’s autoinflammatory gene panel confirmed the presence of a heterozygous variant in the *MEFV* gene (p. Met694Val), a pathogenic mutation causing FMF. To our knowledge, FMF is the first autoinflammatory disease known to be caused by a mutation in the pyrin protein, presenting with monoarticular arthritis, periodic fever, erysipeloid erythema, and abdominal pain. Further genetic analysis confirmed the presence of 5% mosaicism in the *NLRP3* gene (p. Asp305Glu). Germline mutations are known to be the main cause of NOMID; however, our patient had a somatic mutation, which can be present in up to 69% of patients with NOMID who are negative for germline mutations.^5^ These patients usually present with an urticaria-like rash associated with fever, but without any neurologic symptoms.^5^

The onset of flagellate dermatitis varies with its etiology. Bleomycin-induced dermatitis onset usually takes between 1 day to 9 weeks, with a cumulative dose >100 U.^1^ In shiitake mushroom dermatitis, it approximately occurs within 48 hours after consumption.^6^^,^^7^ We hypothesized that the longer duration of onset in our case may be because of a delayed-type hypersensitivity reaction to anakinra.

Flagellate erythema is mostly managed with topical corticosteroids and antihistamines. In cases of bleomycin-induced flagellate dermatitis, cessation of medication may be required if the reaction is severe.^8^ A reported case of flagellate erythema of dermatomyositis in a pediatric patient improved with oral corticosteroids and methotrexate.^2^ There have been no reports so far regarding the use of colchicine in flagellate dermatitis. Colchicine is US Food and Drug Administration-approved antiinflammatory drug used for treating gout and FMF. It acts on multiple pathways, inhibiting microtubule polymerization, impairing neutrophil function and migration, and inhibiting the production of neutrophil superoxide, a key contributor to the inflammatory response seen in neutrophil activation.^9^ It also interrupts granule release in mast cells and decreases levels of the proinflammatory cytokines interleukin (IL)1β , interferon gamma, IL-18, and IL-6.^9^ This may explain the beneficial effect of colchicine on flagellate dermatitis, given the presence of neutrophils in histology.

In the present case, we postulate that the appearance of flagellate-like dermatitis is associated with the anakinra IL-1 receptor antagonist action on the autoinflammatory disorder. Flagellate-like dermatitis responded drastically after the initiation of colchicine; however, the underlying mechanism remains unclear. There are currently no studies on the association between flagellate dermatitis and anakinra.

## Conflicts of interest

None disclosed.
